# Kaempferol Improves Alzheimer's Disease by Inhibiting Neuronal Ferroptosis via Activating GPX4/AKR1C3 Signaling Pathway

**DOI:** 10.1002/prp2.70255

**Published:** 2026-04-30

**Authors:** Le Li, Manying Yang, Jiale Tao, Yonghong Zhao, Na Zhao, Shiguo Sun

**Affiliations:** ^1^ College of Chemistry & Pharmacy Northwest A&F University Yangling Shaanxi China; ^2^ Key Laboratory of Xinjiang Phytomedicine Resource and Utilization, Ministry of Education, Department of Pharmacology Shihezi University Shihezi Xinjiang China

**Keywords:** AKR1C3, aldo‐keto reductase family 1 member B1, Alzheimer's disease, Kaempferol, neuronal ferroptosis

## Abstract

Kaempferol has been shown to be beneficial in the treatment of Alzheimer's disease (AD) in animal models. However, the action mechanism remains unclear. AKR1B1 has been identified as a target of kaempferol, initially suggested by the Therapeutic Target Database, DrugBank, and PubChem, and subsequently confirmed through experimental validation. Kaempferol treatment facilitated the expression of AKR1B1 in PC12 cells exposed to Aβ_1–42_. Kaempferol treatment mitigated the Aβ_1–42_‐induced increases in Fe^2+^, MDA, and lipid ROS and Aβ_1–42_‐induced decreases in GSH synthesis and SOD activity. The reduction in ferroptosis‐related proteins (GPX4, NQO1, SLC7A11, AKR1C1, and AKR1C3) and the inhibition of Nrf2 nuclear translocation and Nrf2/HO‐1 signaling caused by Aβ_1–42_ were also reversed by kaempferol. Overexpressing AKR1B1 led to decreased levels of Fe^2+^, MDA, and lipid ROS, along with increased GSH synthesis and SOD activity in Aβ_1–42_‐treated cells, although these effects were negated by Nrf2 inhibition. The upregulation of GPX4 and AKR1C3 by AKR1B1 overexpression was also reversed when Nrf2 expression was inhibited. Notably, silencing AKR1B1 counteracted the protective effects of kaempferol against Aβ_1–42_‐induced neuronal ferroptosis. In vivo studies revealed that kaempferol improved cognitive impairments, reduced deposition of Aβ and p‐Tau, and alleviated neuronal ferroptosis in the hippocampal tissues of an AD mouse model in a dose‐dependent manner, effects that were diminished by inhibiting AKR1B1 expression. Following kaempferol treatment, the levels of GPX4 and AKR1C3 in the hippocampus of AD mice were found to be reduced. Overall, our findings indicate that kaempferol treatment enhances cognitive function and mitigates pathological alterations in AD mice by inhibiting neuronal ferroptosis through the activation of the Nrf2/HO‐1/GPX4/AKR1C3 signaling via upregulation of AKR1B1. This research supports the need for further investigation and clinical exploration of kaempferol.

**Abbreviations**
AD: Alzheimer's disease
AKR1B1: aldo‐keto reductase family 1 member B1
AKR1C1: aldo‐keto reductase family 1 member C1
AKR1C3: aldo‐keto reductase family 1 member C3
ALOX5: polyunsaturated fatty acid 5‐lipoxygenase
AR: androgen receptor
Aβ: β‐amyloid
CYP1B1: cytochrome P450 1B1
GLO1: glyoxalase I
GPX4: glutathione peroxidase 4
GSH: glutathione
GSSG: oxidized glutathione
HO‐1: heme oxygenase‐1
MDA: malondialdehyde
NQO1: NAD(P)H quinone dehydrogenase 1
Nrf2: erythroid‐derived 2‐related factor 2
SLC7A11: solute carrier family 7 member 11
SOD: superoxide dismutase
TTD: therapeutic target database

## 1 Introduction

Alzheimer's disease (AD) is a progressive neurodegenerative disorder characterized by dysfunctions of memory, language, and thinking, affecting approximately 50 million individuals globally in 2020, with projections suggesting this number could rise to 152 million by 2050. The disease predominantly impacts those over the age of 65 [1, 2]. From a pathological standpoint, AD is characterized by the presence of senile plaques and neurofibrillary tangles, which are caused by β‐amyloid (Aβ) accumulation in the extracellular space and the misfolding and accumulation of tau protein in intracellular spaces, respectively [3]. At present, there is a lack of effective treatments for AD, with only *N*‐methyl‐d‐aspartate antagonists and cholinesterase inhibitors receiving approval for AD treatment [2]. With the development of research associated with AD treatment, multiple AD‐modifying drugs are under development, including Aβ‐targeted drugs (aducanumab and lecanemab) and tau‐targeted drugs (semorinemab and LMTX) [4, 5]. Music therapy, whether used alone or alongside medications and stem‐cell therapy, shows promise as a potential intervention for AD [6, 7].

Beyond the previously mentioned medications and treatment approaches, several significant studies have associated traditional Chinese medicine or its active ingredients with the management of AD. Traditional Chinese medicine and its active ingredients have shown potential in enhancing AD outcomes by influencing various signaling pathways, for instance, NF‐κB, AMPK/mTOR, and erythroid‐derived 2‐related factor 2 (Nrf2)/heme oxygenase‐1 (HO‐1) [8]. Research by Xia Zhao et al. has indicated that ginsenoside RK1, one of the active ingredients of ginseng plants, can alleviate the cognitive impairments and AD‐like pathological changes in animal models and Aβ‐induced PC12 cell damage by activating the AMPK/Nrf2 signaling pathway [9]. In addition, Oxyphylla A, extracted from *Alpinia oxyphylla*, has been found to enhance the cognitive function in the AD murine model and reduce the neuropathological changes in the AD model both in vitro and in vivo by activating AKT/GSK3β and Nrf2/Keap1/HO‐1 signaling pathways [10]. The Nrf2/HO‐1 signaling pathway is vital for the beneficial effects of traditional Chinese medicine and its active ingredients on AD treatment. In our present study, we explored the functions and mechanisms of action of kaempferol, an active ingredient from *Viola tianschanica Maxim*., in alleviating AD‐like cognitive impairments and pathological changes.

In recent years, neuronal ferroptosis has been found to play a crucial regulatory role in AD development. Research by Zheng et al. has demonstrated that artemisinin can improve AD‐like pathological alterations and cognitive deficits in 3× Tg AD mice by inhibiting neuronal ferroptosis. This is achieved through the enhancement of Nrf2 nuclear translocation and the upregulation of expression of both solute carrier family 7 member 11 (SLC7A11) and glutathione peroxidase 4 (GPX4) [11]. SLC7A11 and GPX4 are known inhibitors of ferroptosis [12]. Previous studies have highlighted the importance of exploring methods to inhibit neuronal ferroptosis during AD advancement. In this study, we investigated whether kaempferol can impede AD progression by targeting the Nrf2/HO‐1 signaling pathway to suppress neuronal ferroptosis and elucidated the underlying molecular mechanisms. Our findings may support the potential use of kaempferol as a therapeutic agent for AD.

## 2 Materials and Methods

### 2.1 Data Mining

The promising targets of kaempferol were obtained from three databases, including Therapeutic Target Database (TTD), DrugBank, and PubChem (Table S1). A Venn diagram was employed to analyze the overlapping targets from these sets, which included aldo‐keto reductase family 1 member B1 (AKR1B1), androgen receptor (AR), cytochrome P450 1B1 (CYP1B1), lactoylglutathione lyase (GLO1, also known as glyoxalase I), and polyunsaturated fatty acid 5‐lipoxygenase (ALOX5). Moreover, a protein–protein interaction network was constructed for the kaempferol targets (AKR1B1, AR, CYP1B1, GLO1, and ALOX5) alongside ferroptosis‐associated proteins (GPX4, NQO1, NFE2L2/Nrf2, HMOX1/HO‐1, SLC7A11, AKR1C1 [aldo‐keto reductase family 1 member C1], and AKR1C3 [aldo‐keto reductase family 1 member C3]) using the STRING database.

### 2.2 Cell Culture and Treatment

The rat pheochromocytoma PC12 cell line was sourced from the Center for Excellence in Molecular Cell Science (Research Resource Identifiers catalog number: TCR 9; Cell Resource Center of Shanghai Institute of Biological Sciences, Chinese Academy of Sciences, Shanghai, China). Cells were cultured in complete DMEM medium (Gibco; Grand Island, NY, USA) supplemented with 10% fetal bovine serum (FBS; Sigma‐Aldrich, St. Louis, MO, USA) and 1% penicillin/streptomycin (Sigma‐Aldrich) at a temperature of 37°C. Cells were grown in either cell culture plates or flasks, which were incubated in an environment with 5% CO_2_. Cell culture medium was refreshed every 2 days. Upon reaching 80% confluency, Aβ_1–42_ (10 μM, Ontores Biotechnologies, Hangzhou, China) was introduced to establish an in vitro AD model. Kaempferol was administered at a concentration of 10 μM to the PC12 cells, excluding those used for the MTT assay. The plasmid encoding AKR1B1 and the corresponding AKR1B1 siRNA (si‐AKR1B1, 5′‐AATGCGTGTCTCGAACTTAACAA‐3′) were procured from GeneChem (Shanghai, China) and were transfected into the cells using Lipofectamine 3000 (Invitrogen, CA, USA).

### 2.3 Cell Viability Detection

MTT assay was used to detect the effect of kaempferol on the viability of PC12 cells. Cells (2 × 10^3^ cells/well) were seeded into 96‐well plates, and after a 12‐h incubation, cells were treated with kaempferol at concentrations of 1, 5, and 10 μM, either alone or in combination with Aβ_1–42_ for a duration of 24 h. Following this treatment, the cell culture medium was replaced with 0.5 mg/mL MTT solution dissolved in the same medium (Solarbio, Beijing, China). After a 4‐h incubation period, the MTT solution was removed, and DMSO solution (100 μL) was added to each well to dissolve the blue formazan crystals. Finally, the absorbance of each well was measured at 570 nm using a microplate reader (Bio‐Rad, Hercules, CA, USA).

### 2.4 Measurement of Gene Expression

Total RNA was extracted from brain tissues and PC12 cells using TRIzol reagent (Invitrogen). Then, total RNA served as a template for cDNA synthesis, which was performed with a reverse transcription kit (Takara, Dalian, China). Next, real‐time PCR was conducted using SYBR Green Fast qPCR Mix (Agilent Technologies, Santa Clara, CA, USA) on a designated PCR instrument (Roche Life Science). *GAPDH* acted as the internal reference gene. The relative gene expression was calculated using the 2^−ΔΔ*Ct*^ method. All processes were conducted according to the manufacturer's protocols for the kits or reagents. The primers used in our study are listed in Table S2.

### 2.5 Detection of Protein Expression

Total protein was extracted from brain tissues or PC12 cells using RIPA buffer (Solarbio), and the protein concentrations were measured with a BCA detection kit (Solarbio). For nuclear protein extraction, a Nuclear Protein Extraction kit (Sigma‐Aldrich) was employed. The protein samples were then subjected to separation via SDS‐PAGE gel and subsequently were transferred to a PVDF membrane (0.22 μm; Millipore, Bedford, MA, USA). The membranes were blocked with 5% BSA or 5% nonfat milk for 50–60 min at room temperature. Following this, the membranes were incubated overnight at 4°C with primary antibodies against AKR1B1, Nrf2, p‐Nrf2, HO‐1, GPX4, AKR1C3, and β‐actin (as an internal reference). The primary antibodies, sourced from Abcam (Cambridge, MA, USA), were diluted to a 1:1000 proportion with BSA or nonfat milk. The next day, after washing the membranes three times with 1× TBST, they were incubated with HRP‐conjugated secondary antibodies for 2 h at room temperature. After incubation with ECL reagent (Solarbio), the immunoreactive bands on the membranes were visualized using the Bio‐Rad Gel Doc XR system, and the band intensities were quantified using Image J software.

### 2.6 Analysis of Cell Apoptosis

Twenty‐four hours after Aβ_1–42_ and kaempferol treatment, the effect of kaempferol on the apoptosis of PC12 cells induced by Aβ_1–42_ was assessed using TUNEL staining. The cell culture medium was discarded, and the cells were washed three times with PSB buffer. The cells were fixed with 4% PFA for 30 min. Subsequently, the cells were incubated with a 0.3% Triton X‐100 solution for 5 min. Following this, the TUNEL reaction mixture (50 μL/well; Beyotime, Shanghai, China) was applied. The cells were incubated with the TUNEL reaction mixture at 37°C for 1 h in the dark. At last, the cells were mounted with anti‐fade fluorescence mounting medium (Beyotime), and TUNEL‐positive cells were observed under a fluorescence microscope, and their quantities were analyzed using ImageJ software.

### 2.7 Determination of the Levels of Fe^2+^, Malondialdehyde (MDA), Glutathione/Oxidized Glutathione (GSH/GSSG), and Superoxide Dismutase (SOD)

Intracellular iron concentration in PC12 cells was measured utilizing the Ferrous Ion Content Assay Kit (Solarbio), and the iron concentration in brain tissues was examined using the Tissue Iron Content Assay Kit (Solarbio). The MDA level was assessed using the MDA Assay Kit (Solarbio). The ratio of reduced GSH/GSSG was examined using the GSH/GSSG Ratio Detection Assay Kit (Abcam). The SOD level was determined using the SOD Activity Assay Kit (Solarbio). All detections were conducted according to the manufacturer's protocols.

### 2.8 Measurement of Total ROS and Lipid ROS Levels

To assess the total intracellular levels of ROS, PC12 cells were cultured in six‐well plates and subsequently treated with the designated drugs. Twenty‐four hours later, the cells were washed with saline and incubated with 10 μM DCFH‐DA reagent (diluted in DMEM medium; Beyotime) at 37°C for 1 h. Finally, the stained cells were observed under a fluorescence microscope with an excitation wavelength of 488 nm, and the fluorescence intensities of the cells were quantified using ImageJ software. For evaluating lipid ROS levels, the BODIPY 581/591 C11 staining reagent (ThermoFisher Scientific, Waltham, MA, USA) was employed. Twenty‐four hours after drug treatment, the cells were washed with saline and incubated with 2.5 μM BODIPY 581/591 C11 reagent at 37°C for 30 min. Similarly, the stained cells were observed under a fluorescence microscope, and the fluorescence intensities of the cells were analyzed using ImageJ software.

### 2.9 Animal Experiments

C57BL/6J mice (male, 8 weeks old) were purchased from Shanghai Lingchang Biotech Co. Ltd. (Shanghai, China). All animal experiments were conducted with the approval of the Animal Experimentation Ethics Committee of Shihezi University and based on the ARRIVE guidelines.

Mice were raised in a standard environment with ad libitum access to food and water. After acclimatization for 7 days, mice were divided into the Ctrl, Model, Model +30 mg/kg kaempferol, Model +60 mg/kg kaempferol, Kaempferol + AAV‐shNC, and Kaempferol + AAV‐shAKR1B1 groups (*N* = 6). For the AD (model) and treatment groups, mice were injected with Aβ_1–42_ peptides (A9810, Sigma, Missouri, USA) to establish a validated AD model. In brief, Aβ_1–42_ peptide powder was dissolved in sterile distilled water (1 mg/mL) to form Aβ_1–42_ oligomer for 3 days at room temperature. Then, mice were anesthetized with isoflurane, and stereotaxic surgery was performed. Upon craniotomy being conducted, 5 μL of Aβ_1–42_ solution was injected into the hippocampus region of mice through a Hamilton syringe. Adeno‐associated virus (AAV) expressing the AKR1B1‐specific shRNA was purchased from Genomeditech (Shanghai, China). The coordinates of injection were 0.5 mm posterior to the bregma and 1.0 mm lateral to the sagittal suture. Mice were injected with penicillin daily for the first 4 days after surgery to prevent infection. For the Ctrl group, mice underwent the same procedures, but the Aβ_1–42_ peptide solution was replaced with an equivalent volume of sterile distilled water. Four days after the injection of Aβ_1–42_ peptides or saline, some mice in the model group were treated with 30 or 60 mg/kg kaempferol daily by gavage for 30 days. For Kaempferol + AAV‐shNC and Kaempferol + AAV‐shAKR1B1 groups, mice received kaempferol treatment (60 mg/kg) and were injected unilaterally at a rate of 0.1 μL/min with AAV expressing negative control shRNA or AAV expressing AKR1B1 shRNA (1 μL, 7.5 × 10^12^ viral particles per mL) through a microinjection system.

### 2.10 Behavioral Tests

The Morris water maze is a commonly utilized method for studying the psychological processes and neural mechanisms of spatial learning and memory. The apparatus of the Morris water maze test consists of a circular pool (35 cm height and 1.5 m diameter) (XR‐XM101, Shanghai Xinruan Information Technology Co. Ltd., Shanghai, China) and a fixed security platform in the pool. Water at around 25°C was added to the pool, and the horizontal plane was 2 cm higher than the platform. The mice received four habituation training sessions on Day 0. Then, tests were conducted for five consecutive days. All the data were recorded automatically through a video tracking system. The escape latency times in crossing the original platform and the time in the target quadrant of mice were analyzed.

The novel object recognition test was fulfilled utilizing the SAMRT V3.0 (Pan Lab, Barcelona, Spain), aimed at analyzing the learning and memory abilities of mice. The exploration time of the object was defined as the time the mice spent with their noses within 2 cm of the object. Novel object index (NOI) (%) = [(Novel object exploration‐familiar object exploration time)/(Novel exploration time + familiar object exploration)] × 100%.

### 2.11 Immunohistochemistry

Immunohistochemical analysis was conducted to assess the expression levels of Aβ and p‐Tau (S396) in mouse brain tissues. The tissue sections underwent a process of deparaffinization and rehydration, followed by antigen retrieval using a target retrieval solution in a boiling water bath for 10 min. Subsequently, the sections were incubated with 0.3% hydrogen peroxide solution (H_2_O_2_) for 30 min to inhibit any endogenous peroxidase activity. Next, the sections were blocked with 5% normal goat serum for 1 h at room temperature and then incubated overnight at 4°C with primary antibodies against Aβ (1:800 dilution; Bioss, Beijing, China) and p‐Tau (S396) (1:1000 dilution; Abcam). The next day, the sections were incubated with biotin‐conjugated goat anti‐rabbit IgG antibody, and finally, they were incubated with DAB solution. The sections were observed with a light microscope using an optical microscope (Olympus, Tokyo, Japan).

### 2.12 ELISA Assay

The concentrations of TNF‐α, IL‐6, and IL‐1β in the hippocampal tissues were measured using ELISA kits (Shanghai Lianke Biological Co. Ltd., Shanghai, China).

### 2.13 Statistical Analysis

All statistical evaluations were performed using SPSS software, version 25.0 (IBM, USA), and GraphPad Prism version 9.0 (GraphPad, USA). The data are presented as the mean ± standard deviation (SD). Statistical significance was ensured by Student's *t*‐test (two independent groups) and one‐way ANOVA followed by Tukey's test (multiple comparisons). A *p* value < 0.05 was considered to indicate statistical significance. Each experiment was measured with at least three replicates.

## 3 Results

### 3.1 AKR1B1 Was One of the Potential Targets of Kaempferol

**FIGURE 1:**
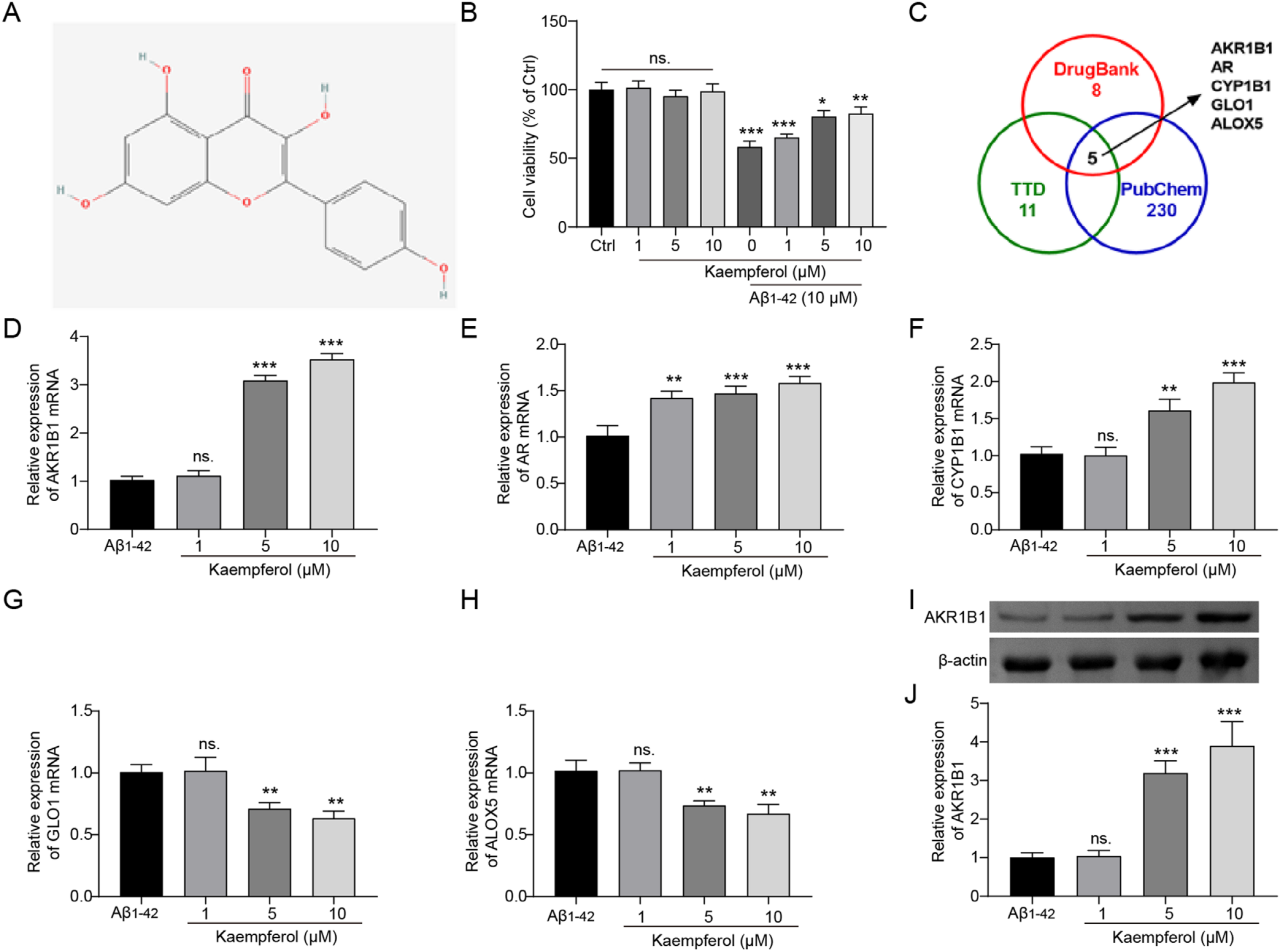
AKR1B1 was a potential target of kaempferol. (A) The 2D structure of kaempferol. (B) 1, 5, and 10 μM kaempferol were utilized to treat the PC12 cells alone or in combination with 10 μM Aβ_1–42_. Cell viability was measured by the MTT assay. (C) TTD, DrugBank, and PubChem databases were used to excavate and integrate the potential targets of kaempferol, and the intersections of the three sets were processed utilizing a Venn diagram. Aβ_1–42_‐treated PC12 cells were administered with 1, 5, and 10 μM kaempferol. (D–H) The mRNA expression levels of AKR1B1, AR, CYP1B1, GLO1, and ALOX5 were detected by RT‐qPCR. (I–J) A Western blot was conducted to evaluate the expression of AKR1B1 protein. *p < 0.05, **p < 0.01, and ***p < 0.001. All experiments were conducted at least three times independently.

According to the previous analysis, we focused on the role and the mechanism of action of kaempferol in improving AD. The 2D structure of kaempferol was displayed in Figure 1A. Our results demonstrated that varying doses of kaempferol, ranging from 0 to 10 μM, have no effect on the viability of PC12 cells, while kaempferol treatment significantly enhanced the cell viability following Aβ_1–42_ exposure in a dose‐dependent manner (Figure 1B). To identify the potential targets of kaempferol, we sourced data from three databases, including TTD, DrugBank, and PubChem. The intersection among these datasets revealed targets, including AKR1B1, AR, CYP1B1, GLO1, and ALOX5 (Figure 1C). We detected the expression levels of these proteins in PC12 cells treated with Aβ_1–42_ to evaluate how kaempferol regulates them under conditions mimicking AD. Our results showed that treatment with kaempferol at concentrations of 5 and 10 μM significantly increased AKR1B1 mRNA levels (Figure 1D). AR mRNA expression was also promoted by kaempferol treatment in a dose‐dependent manner from 1 to 10 μM (Figure 1E). A volume of 5 and 10 μM doses of kaempferol facilitated CYP1B1 mRNA expression (Figure 1F). Conversely, 5 and 10 μM doses of kaempferol inhibited the expression of both GLO1 mRNA and ALOX5 mRNA (Figure 1G,H). Among them, the most pronounced change was observed in AKR1B1 mRNA levels. We also confirmed the increase in AKR1B1 protein levels following kaempferol treatment in Aβ_1–42_‐treated PC12 cells (Figure 1I,J). Based on the above experiments, we identified AKR1B1, AR, CYP1B1, GLO1, and ALOX5 as potential targets of kaempferol, with a particular emphasis on AKR1B1 for our subsequent investigations.

### 3.2 Kaempferol Suppressed Aβ_1–42_ ‐Induced Ferroptosis of PC12 Cells Through Nrf2/HO‐1 Signaling Pathway

**FIGURE 2:**
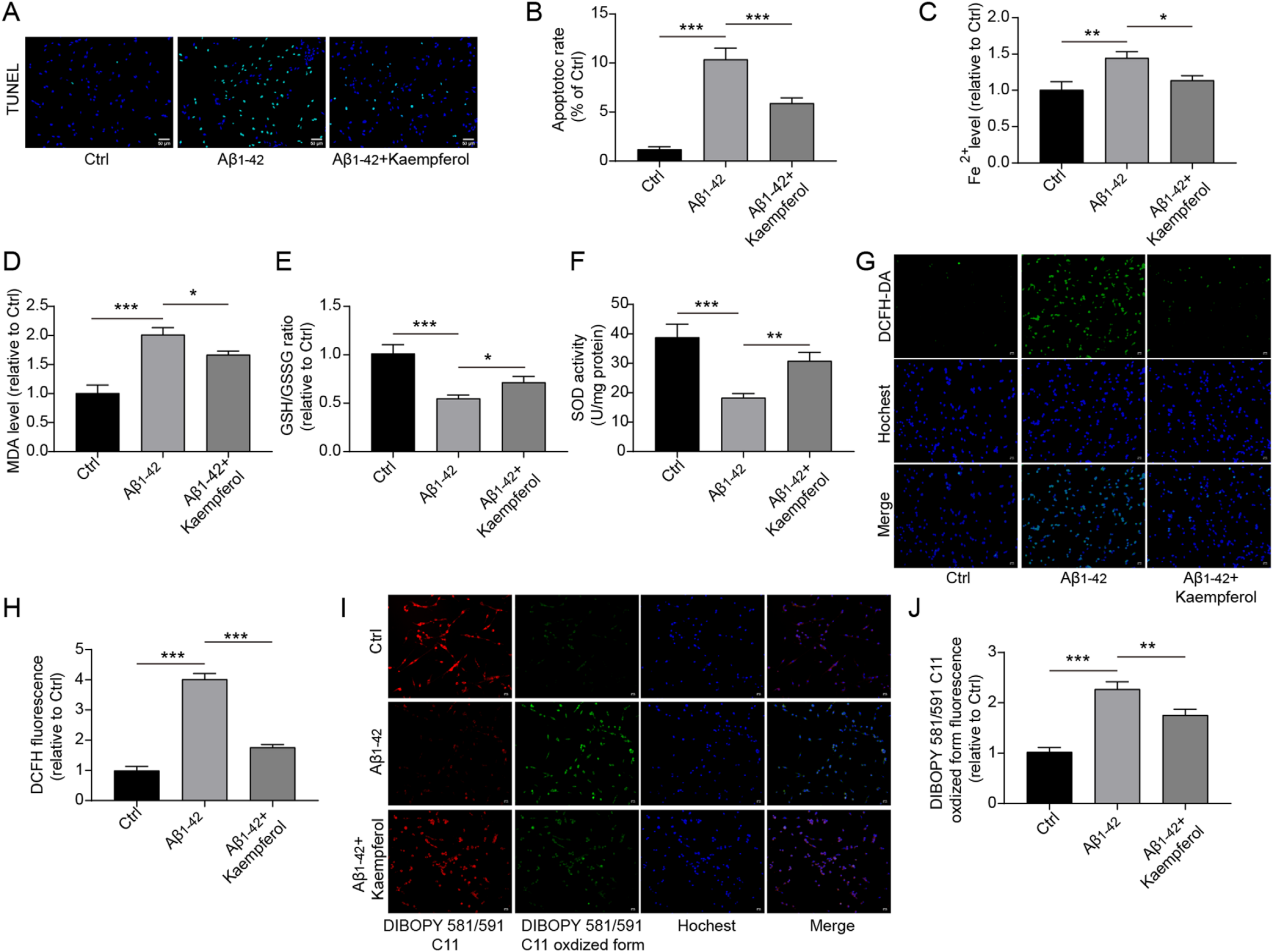
Kaempferol suppressed Aβ_1–42_‐induced deposition of lipid ROS in PC12 cells. Cells were treated with Aβ_1–42_ (10 μM) alone or in combination with kaempferol (10 μM). (A and B) TUNEL staining was conducted to detect cell apoptosis. (C) The level of Fe^2+^ in cells was examined using an iron detection kit. (D) The level of MDA was examined using an MDA detection kit. (E) The GSH synthesis detection kit was utilized to detect the ratio of GSH/GSSG. (F) The SOD activity detection kit was used to determine the level of SOD. (G–J) The accumulation of total ROS and lipid ROS was determined by DCFH‐DA staining and BODIPY 581/591 C11 staining, respectively. *p < 0.05, **p < 0.01, and ***p < 0.001. All experiments were repeated at least three times.

In order to investigate the action mechanism by which kaempferol exerts its effects, we detected the levels of factors associated with cell apoptosis and ferroptosis. Treatment with kaempferol was found to reduce the apoptosis induced by Aβ_1–42_ in PC12 cells (Figure 2A,B). The concentration of Fe^2+^ in PC12 cells exposed to Aβ_1–42_ was elevated, but this increase was significantly diminished with kaempferol treatment (Figure 2C). Following Aβ_1–42_ administration, there was an increase in the MDA level, alongside a decrease in the GSH/GSSG ratio and SOD activity in PC12 cells, which were partially restored by kaempferol treatment (Figure 2D–F). In addition, the results of DCFH‐DA staining showed that the content of intracellular ROS in Aβ_1–42_‐treated PC12 cells was evaluated, which was also reversed by kaempferol treatment (Figure 2G,H). A significant accumulation of lipid ROS was observed in Aβ_1–42_‐treated PC12 cells, but kaempferol treatment could reduce the accumulation of lipid ROS (Figure 2I,J). Therefore, we propose that kaempferol may inhibit ferroptosis in PC12 cells induced by Aβ_1–42_.

**FIGURE 3:**
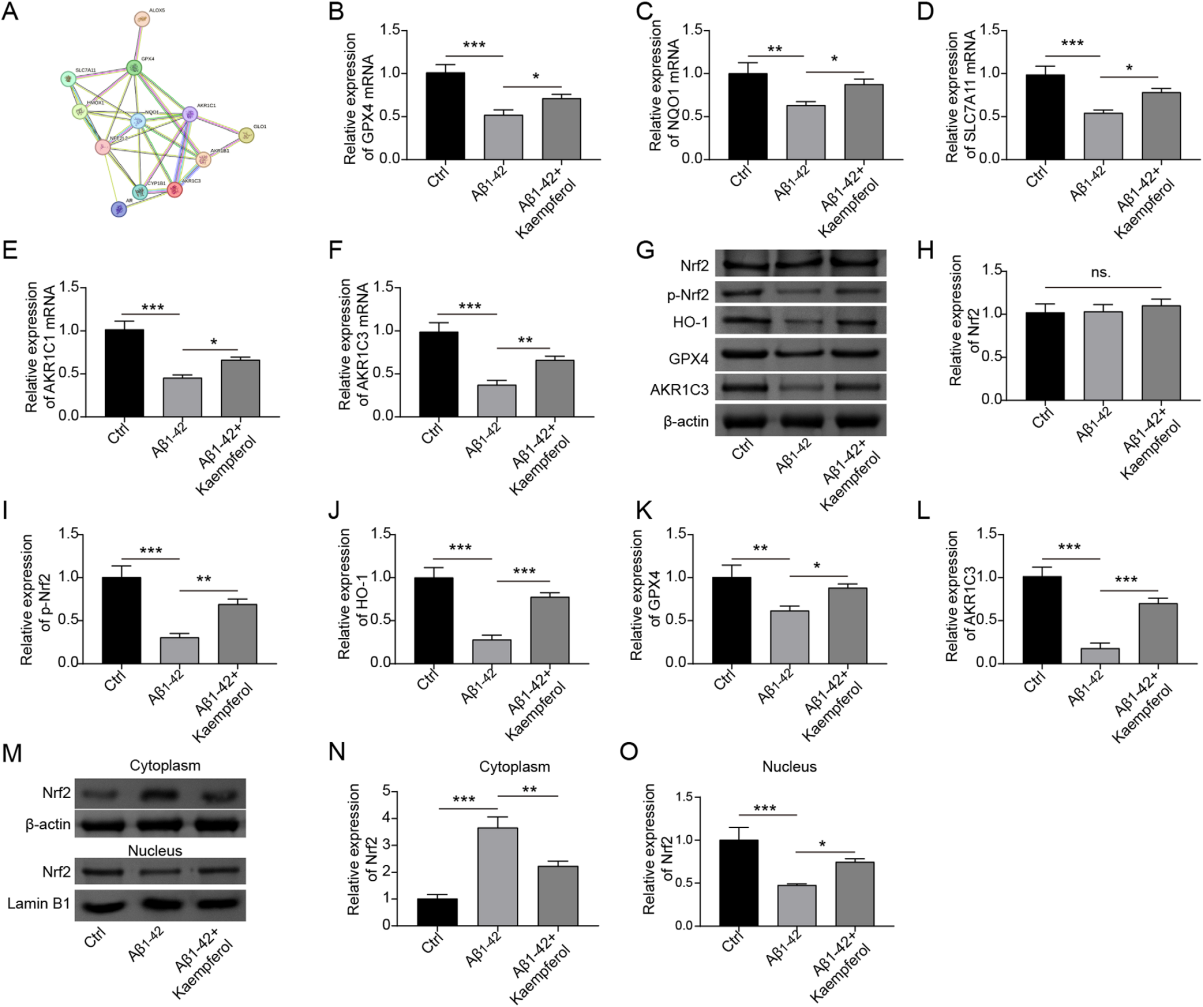
Kaempferol suppressed Aβ_1–42_‐induced ferroptosis of PC12 cells. (A) The interrelation among AKR1B1, AR, CYP1B1, GLO1, ALOX5, and ferroptosis‐related markers (GPX4, NQO1, NFE2L2/Nrf2, HMOX1/HO‐1, SLC7A11, AKR1C1, and AKR1C3) was analyzed utilizing the STRING database. PC12 cells were treated with Aβ_1–42_ (10 μM) alone or in combination with kaempferol (10 μM). Then, (B–F) RT‐qPCR was carried out to detect the expression of GPX4, NQO1, SLC7A11, AKR1C1, and AKR1C3 at the mRNA level. (G–L) Western blot was performed to detect the expression of Nrf2, phosphorylated Nrf2, HO‐1, GPX4, and AKR1C3 at the protein level. (M and N) Protein samples were extracted from the nucleus and cytoplasm to determine the Nrf2 level using a Western blot. *p < 0.05, **p < 0.01, and ***p < 0.001. All experiments were repeated at least three times.

We further analyzed the connections between the above targets of kaempferol (AKR1B1, AR, CYP1B1, GLO1, and ALOX5) and ferroptosis‐related proteins (GPX4, NQO1, NFE2L2/Nrf2, HMOX1/HO‐1, SLC7A11, AKR1C1, and AKR1C3) utilizing the STRING tool, revealing intricate interactions (Figure 3A). Aβ_1–42_ treatment resulted in a decrease in the mRNA levels of GPX4, NQO1, SLC7A11, AKR1C1, and AKR1C3, but the levels of the above mRNAs were increased following the administration of kaempferol (Figure 3B–F). The inhibition of Aβ_1–42_ to the activation of Nrf2 and the inhibition of Aβ_1–42_ to the protein expression levels of HO‐1, GPX4, and AKR1C3 were also rescued by the administration of kaempferol (Figure 3G–L). In the PC12 cells treated with Aβ_1–42_, Nrf2 level was downregulated in the nucleus but was upregulated in the cytoplasm; however, kaempferol treatment facilitated the nuclear translocation of Nrf2 (Figure 3M–O). In summary, we suggest that kaempferol may suppress Aβ_1–42_‐induced ferroptosis in PC12 cells by activating the Nrf2/HO‐1 signaling pathway.

### 3.3 AKR1B1 Suppressed the Ferroptosis of PC12 Cells by Activating the Nrf2/HO‐1 Signaling Pathway

**FIGURE 4:**
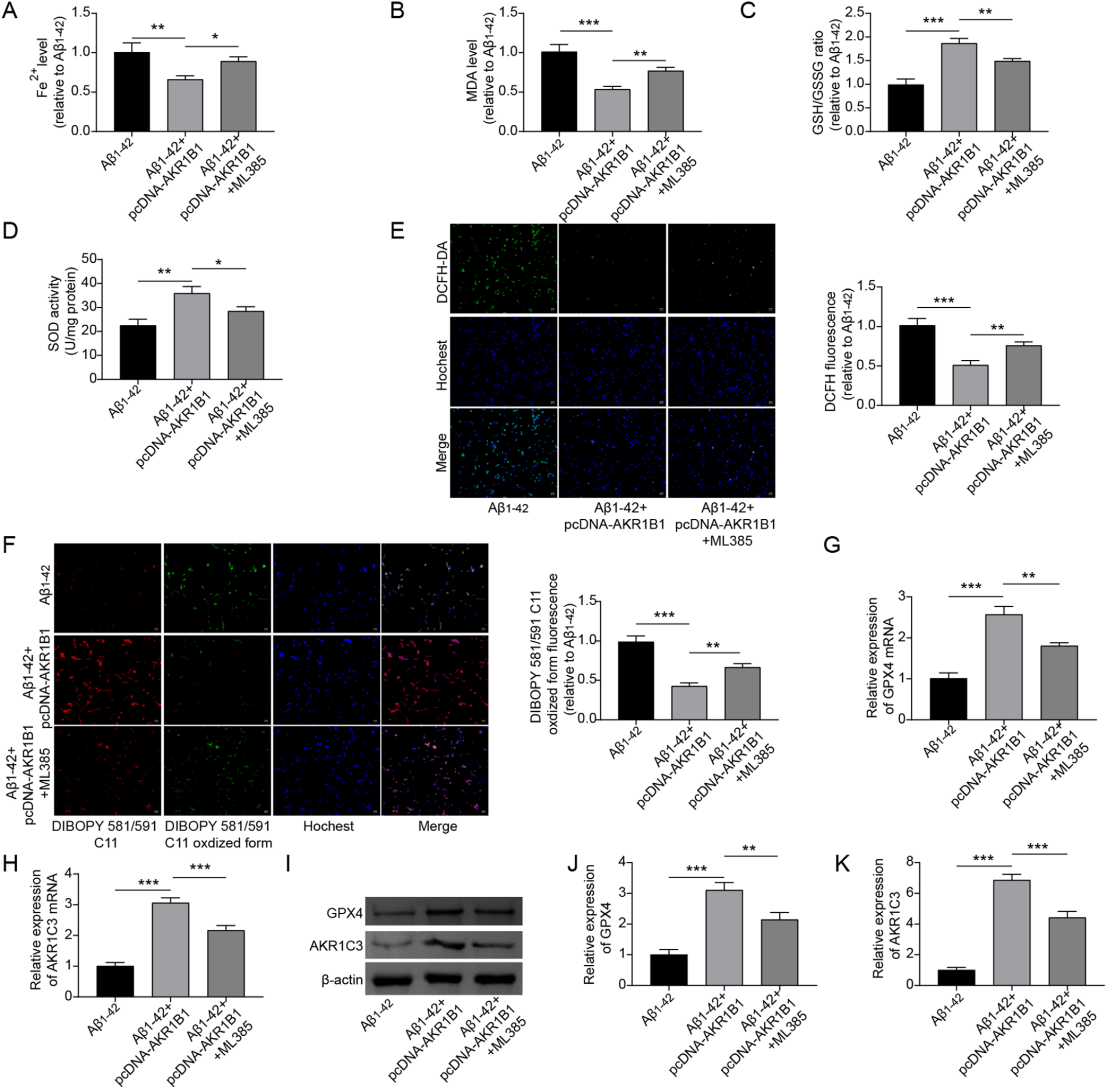
AKR1B1 inhibited Aβ_1–42_‐induced ferroptosis of PC12 cells via activating the Nrf2/HO‐1 signaling pathway. Aβ_1–42_‐induced PC12 cells were transfected with the plasmid expressing AKR1B1 alone or in combination with ML385 treatment. ML385 is an inhibitor of Nrf2. (A–D) The Fe^2+^ level, MDA level, GSH/GSSG ratio, and SOD activity were measured using specific kits. (E and F) The accumulation of total ROS and lipid ROS was determined by DCFH‐DA staining and BODIPY 581/591 C11 staining. (G–H) The mRNA expression of GPX4 and AKR1C3 was measured by RT‐qPCR. (I–K) The expression of GPX4 and AKR1C3 at the protein level was determined by Western blot. *p < 0.05, **p < 0.01, and ***p < 0.001. All experiments were repeated at least three times.

In order to investigate whether AKR1B1 is involved in Aβ_1–42_‐induced ferroptosis in PC12 cells via the Nrf2/HO‐1 signaling pathway, we transfected the plasmid encoding AKR1B1 into Aβ_1–42_‐induced PC12 cells. Our findings indicated that the elevated expression of AKR1B1 led to a significant decrease in the levels of Fe^2+^ and MDA, an effect that was counteracted by Nrf2 inhibition (Figure 4A,B). Increasing AKR1B1 expression upregulated the ratio of GSH/GSSG and the activity of SOD, both of which were rescued upon Nrf2 suppression (Figure 4C,D). Moreover, increasing AKR1B1 expression resulted in lower intracellular ROS levels and reduced lipid ROS deposition in Aβ_1–42_‐treated PC12 cells, whereas Nrf2 inhibition markedly elevated both ROS production and lipid ROS deposition (Figure 4E,F). The protein interaction network shown in Figure 3A highlighted a close association among GPX4, AKR1C3, and AKR1B1. Our experiments further confirmed that increasing AKR1B1 expression led to increased mRNA and protein levels of GPX4 and AKR1C3 in Aβ_1–42_‐treated PC12 cells, with these effects reversed by Nrf2 inhibition (Figure 4G–K). Overexpression of AKR1B1 markedly restrained Aβ_1–42_‐induced ferroptosis by activating the Nrf2/HO‐1 signaling pathway.

### 3.4 AKR1B1 Mediated the Inhibition of Kaempferol on Ferroptosis of PC12 Cells

**FIGURE 5:**
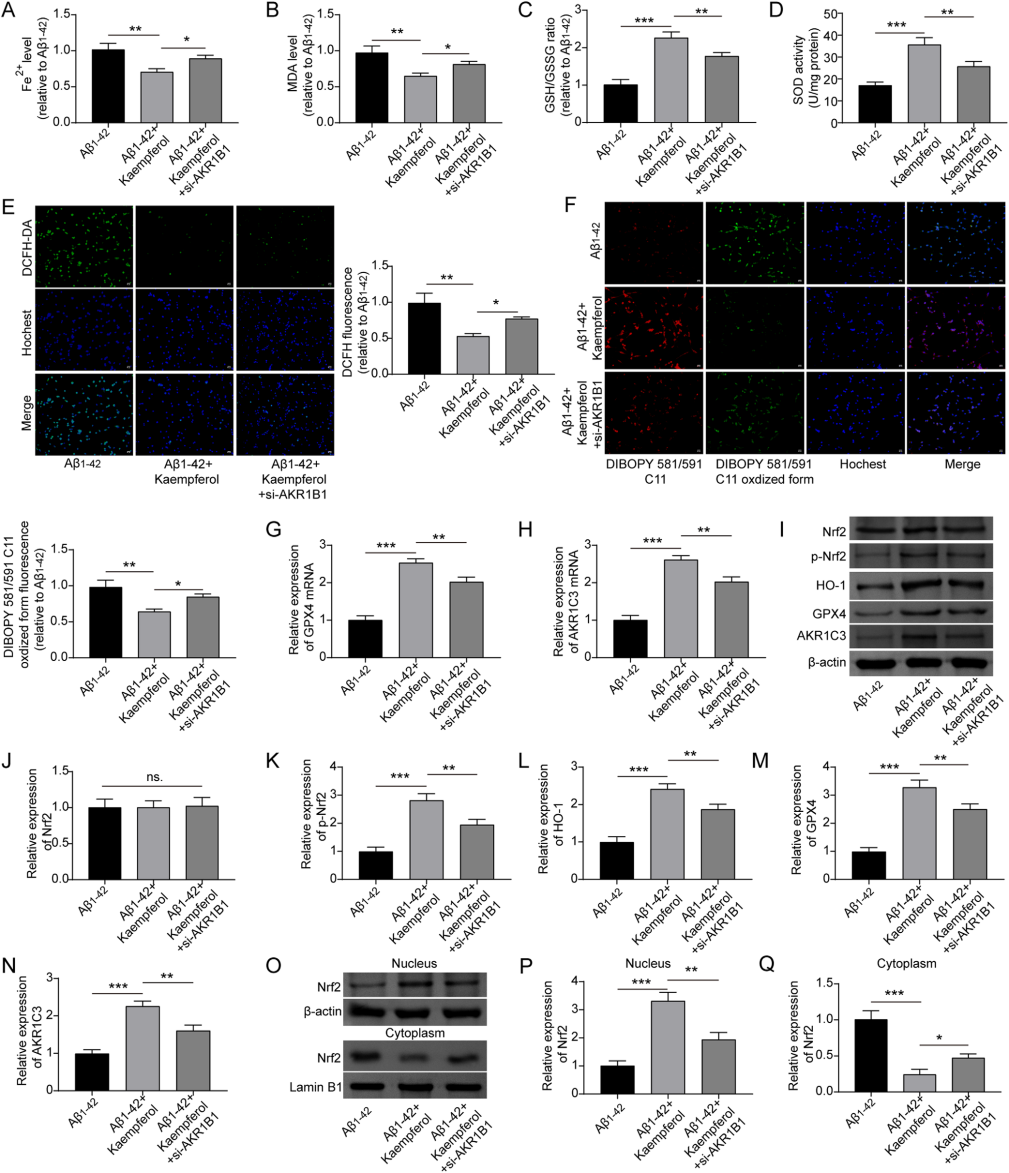
Kaempferol inhibited Aβ_1–42_‐induced ferroptosis via promoting AKR1B1 expression. Aβ_1–42_‐induced PC12 cells were treated with kaempferol alone or in combination with si‐AKR1B1 transfection. (A–D) Fe^2+^ level, MDA level, GSH/GSSG ratio, and SOD activity were determined utilizing specific kits. (E and F) The accumulation of total ROS and lipid ROS was respectively determined by DCFH‐DA staining and BODIPY 581/591 C11 staining, respectively. (G and H) The expression of GPX4 mRNA and AKR1C3 mRNA was measured by RT‐qPCR. (I–N) The expression of GPX4 and AKR1C3 at the protein level and the expression of HO‐1, Nrf2, and phosphorylated Nrf2 were examined by Western blot. (O and P) Protein samples were extracted from the cell nucleus and cytoplasm and were utilized to analyze the level of Nrf2 through Western blot. *p < 0.05, **p < 0.01, and ***p < 0.001. All experiments were repeated at least three times.

To verify whether AKR1B1 mediates the protective effects of kaempferol against Aβ_1–42_‐induced ferroptosis in PC12 cells, the cells were subjected to treatment with AKR1B1 siRNA alongside kaempferol. The reduction in Fe^2+^ level and MDA levels caused by kaempferol treatment in Aβ_1–42_‐induced PC12 cells was partly reversed by the silencing of AKR1B1 (Figure 5A,B). The increase in the GSH/GSSG ratio and SOD activity induced by kaempferol treatment was also diminished when AKR1B1 was silenced (Figure 5C,D). As expected, the inhibition of ROS production and lipid ROS accumulation by kaempferol treatment was reversed upon silencing AKR1B1 (Figure 5E,F). Silencing AKR1B1 remarkably downregulated the promotion of kaempferol to GPX4 mRNA and AKR1C3 mRNA expression (Figure 5G,H). According to the results of Western blotting analysis, the promotory influences of kaempferol on both Nrf2 activation and the expression of HO‐1, GPX4, and AKR1C3 were negated by the silencing of AKR1B1 (Figure 5I–N). In comparison to Aβ_1–42_‐treated PC12 cells, kaempferol treatment facilitated the nuclear translocation of Nrf2, which was partly hindered following AKR1B1 silencing (Figure 5O–Q). In summary, kaempferol inhibited the ferroptosis of PC12 cells induced by Aβ_1–42_ by activating the Nrf2/HO‐1 signaling pathway through the upregulation of AKR1B1 expression.

### 3.5 Kaempferol Ameliorated AD‐Like Pathology in Mouse Model

**FIGURE 6:**
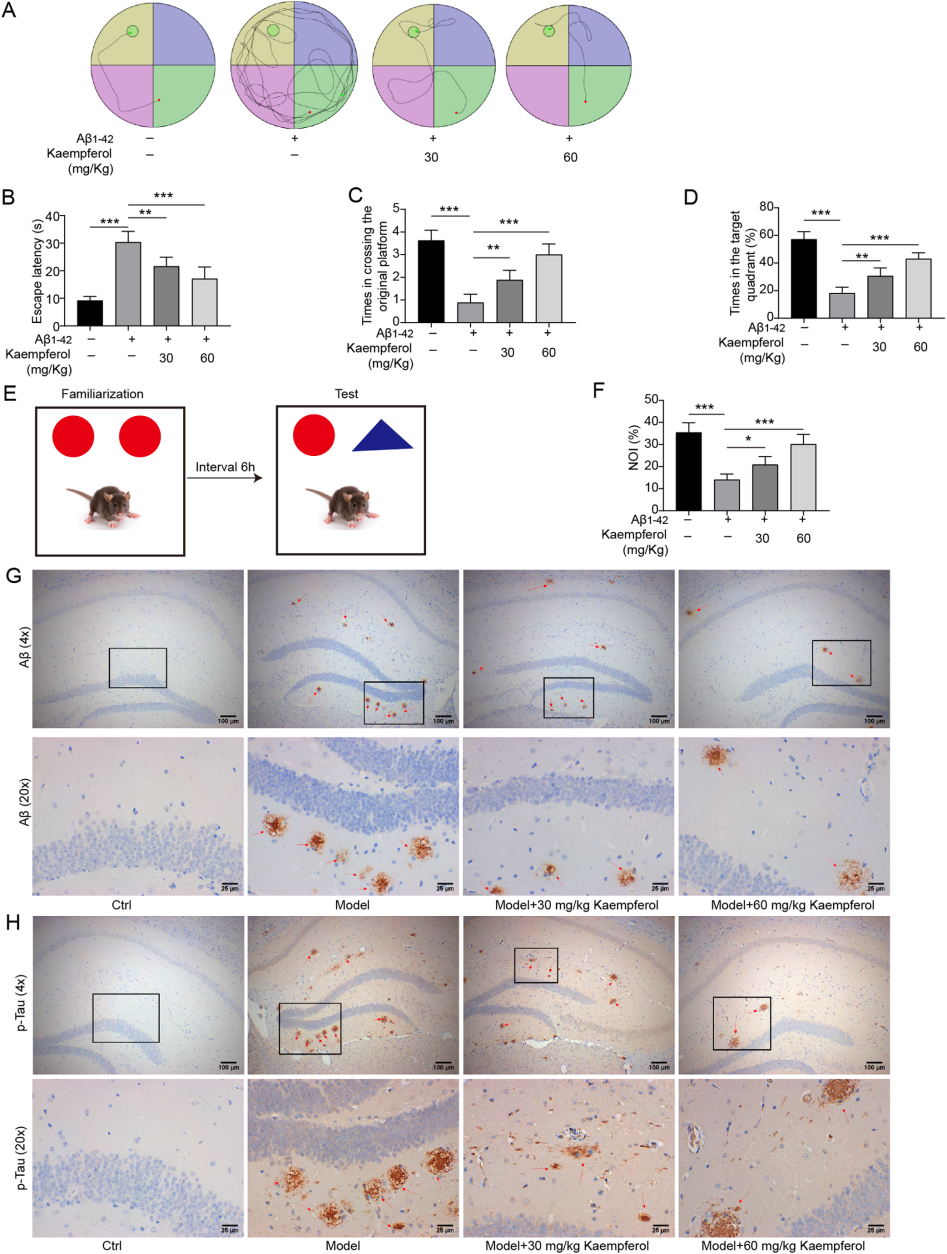
Kaempferol improved the cognitive deficits and neuronal damage of AD mice. Aβ_1–42_‐induced AD model mice were treated with 30 or 60 mg/kg/day kaempferol for 30 days. (A–D) The learning and memory abilities of mice were tested by the Morris water maze test. (E and F) The ability to find novel things in mice was measured by the novel object recognition test analysis. (G and H) The deposition of Aβ and p‐Tau in hippocampal tissues was detected by immunohistochemistry. *p < 0.05, **p < 0.01, and ***p < 0.001. All experiments were repeated at least three times.

We subsequently confirmed the beneficial effects of kaempferol on AD pathology in vivo. Kaempferol administration evidently shortened the escape latency of the AD model mouse and increased both the time in crossing the original platform and the time in the target quadrant of the AD model mouse. A dosage of 60 mg/kg of kaempferol proved to be more effective than 30 mg/kg in improving the cognitive impairment in AD model mice (Figure 6A–D). In addition, the capacity of the AD model mouse to explore novel things was obviously enhanced after kaempferol administration (Figure 6E,F). Immunohistochemical staining showed that kaempferol treatment significantly reduced the deposition of both Aβ (Figure 6G) and p‐tau (Figure 6H) in the hippocampal region, and these effects were more pronounced at higher doses of kaempferol.

**FIGURE 7:**
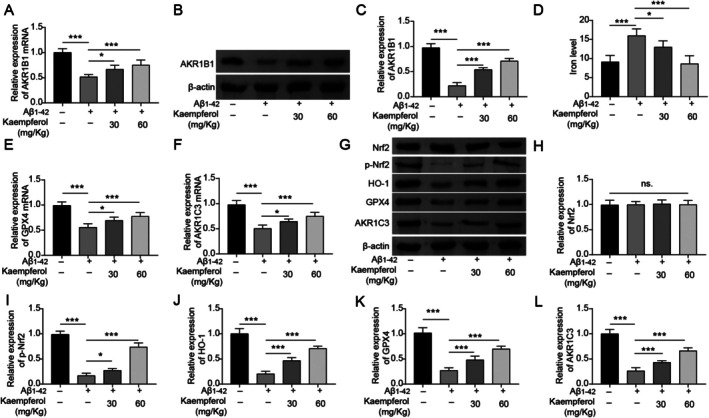
Kaempferol inhibited ferroptosis in the hippocampal tissue of AD mice. Aβ_1–42_‐induced AD model mice were treated with 30 or 60 mg/kg/day kaempferol for 30 days. (A) The expression of AKR1B1 mRNA in hippocampus tissue was examined by RT‐qPCR. (B and C) The expression of AKR1B1 at the protein level was determined by Western blot. (D) The content of iron in hippocampus tissue was examined. (E and F) The expression of GPX4 mRNA and AKR1C3 mRNA in hippocampus tissue was examined by RT‐qPCR. (G–L) Western blot was performed to detect the expression of GPX4, AKR1C3, Nrf2, phosphorylated Nrf2, and HO‐1 in hippocampus tissue at the protein level. *p < 0.05, **p < 0.01, and ***p < 0.001. All experiments were repeated at least three times.

The mRNA and protein levels of AKR1B1 in the hippocampus tissues of AD mice were found to be reduced, while treatment with kaempferol significantly facilitated the expression of AKR1B1 (Figure 7A–C). Consistent with cellular experiments, there was an increase in iron level within the hippocampus tissues of AD mice, and treatment with kaempferol led to a decrease in iron deposition (Figure 7D). The expression levels of GPX4 mRNA and AKR1C3 mRNA in the hippocampus tissues of AD mice were downregulated, while kaempferol treatment facilitated their expression (Figure 7E,F). At the protein level, the expression of GPX4, AKR1C3, phosphorylated Nrf2, and HO‐1 was decreased in the hippocampus tissues of AD mice, while treatment with kaempferol effectively facilitated the expression of these proteins in a dose‐dependent manner (Figure 7G–L). In summary, kaempferol effectively reduced both the behavioral and pathological symptoms associated with AD in mice.

### 3.6 Kaempferol Ameliorated AD‐Like Pathology via Targeting AKR1B1

**FIGURE 8:**
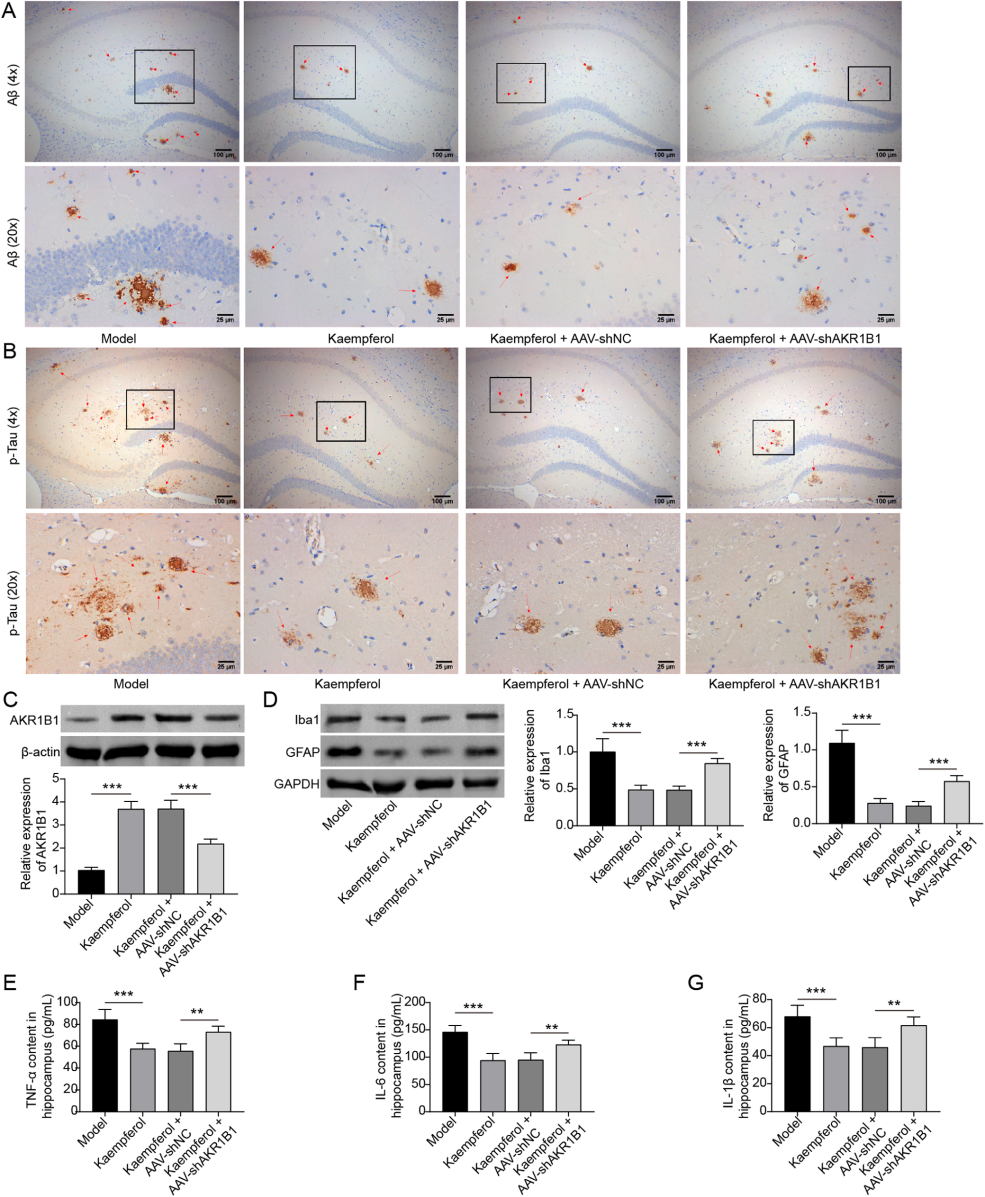
Kaempferol improved the deposition of Aβ and p‐Tau in the hippocampus tissue of AD mice via targeting AKR1B1. A part of the AD mouse model accepted both the kaempferol treatment and the intracranial injection of the AAV virus expressing shRNA specific for AKR1B1. (A) The deposition of Aβ in hippocampal tissues was detected by immunohistochemistry. (B) The deposition of p‐Tau in hippocampal tissues was also detected by immunohistochemistry. (C) The expression of AKR1B1 in hippocampal tissues was measured by Western blot. (D) A Western blot was performed to detect the expression of Iba1 and GFAP. (E–G) The levels of inflammatory factors (TNF‐α, IL‐6, and IL‐1β) were measured by ELISA assay. *p < 0.05, **p < 0.01, and ***p < 0.001. All experiments were repeated at least three times.

To determine if kaempferol alleviates AD‐like pathology in mice by targeting AKR1B1, the AAV carrying AKR1B1 shRNA was used to inhibit AKR1B1 expression. As shown in Figure 8A,B, kaempferol treatment obviously reduced the deposition of both Aβ and p‐Tau in the hippocampus tissues of AD mice, an effect that was counteracted by the inhibition of AKR1B1. Kaempferol treatment induced an increase in AKR1B1 expression, but injection of the AAV expressing AKR1B1 shRNA significantly inhibited the expression of AKR1B1 in the hippocampus tissue of AD mice (Figure 8C). GFAP and Iba1 are well‐known gliosis markers. Our data demonstrated that kaempferol treatment significantly inhibited the expression of both GFAP and Iba1 in the hippocampus tissue of AD mice, whereas the suppression of AKR1B1 expression restored their levels (Figure 8D). Additionally, the expression of inflammatory factors also differed among the four groups. Kaempferol treatment reduced the production of TNF‐α, IL‐6, and IL‐1β in the hippocampus tissues of AD mice, with this reduction being reversed by the inhibition of AKR1B1 (Figure 8E–G). Therefore, we propose that kaempferol improves AD‐like pathology in mice by promoting the expression of AKR1B1.

## 4 Discussion

In our current study, we aimed to explore the potential of kaempferol and its regulatory effects on cognitive deficits and neuronal damage in the AD model. Our findings indicated that kaempferol administration obviously inhibited Aβ‐induced ferroptosis in PC12 cells and alleviated cognitive deficits, tau‐related pathology, and neuroinflammation in the AD mouse model. The underlying mechanism revealed that kaempferol exerts the above functions by targeting AKR1B1 and its downstream Nrf2/HO‐1/GPX4/AKR1C3 signaling pathway. Our findings indicate that kaempferol is a promising drug candidate for AD treatment by combinatorial targeting of tau and Aβ.

Numerous studies have confirmed the beneficial effects of traditional Chinese medicine in the treatment of AD. Kaempferol, one of the active ingredients derived from *V. thianschanica Maxim*., has emerged as a promising candidate for AD therapy [13, 14]. Our research supports this assertion. In Aβ_1–42_‐induced PC12 cells, treatment with kaempferol significantly enhanced cell viability. In an Aβ_1–42_‐induced mouse model of AD, kaempferol administration effectively improved spatial learning ability, memory ability, and the ability to explore new things, while also reducing neuronal damage in the hippocampal tissues of AD mice. AD is characterized by the deposition of extracellular Aβ plaques and intracellular neurofibrillary tangles formed by tau aggregates. Tau is predominantly expressed in neuronal axons with a primary function of promoting the assembly and stability of microtubules. During the progression of AD, hyperphosphorylation of tau is one of the earliest and continuous events. Our data showed that kaempferol treatment significantly reduced the deposition of Aβ and p‐Tau in the hippocampus tissues of AD mice. To further investigate the potential targets of kaempferol, three databases were utilized, including TTD, DrugBank, and PubChem. AKR1B1, AR, CYP1B1, GLO1, and ALOX5 were found to be the targets of kaempferol. Our experiments proved that kaempferol treatment promoted the expression of AKR1B1, AR, and CYP1B1, but it suppressed the expression of GLO1 and ALOX5, with AKR1B1 showing the most pronounced change. Therefore, AKR1B1 emerged as a significant focus in our research.

The majority of studies concerning AKR1B1 have concentrated on its role in the advancement of cancer, such as hepatocellular carcinoma [15]. Before our current study, there were no studies addressing the involvement of AKR1B1 in the development of AD. A few earlier studies indicated that AKR1B1 might regulate ferroptosis. For instance, Dai et al. and colleagues proved the inhibition of AKR1B1 to cancer cell ferroptosis in the progression of gastric cancer by activating SLC7A11 through its interaction with STAT3 [16]. Huifang Pang and others have verified that the decrease of AKR1B1 induced by EPS15‐AS1 overexpression resulted in an enhancement in cancer cell ferroptosis during the development of hepatocellular carcinoma. It is well established that neuronal ferroptosis significantly contributes to neuronal damage in AD development. Therefore, we speculated that kaempferol exerts its effects, at least in part, through ferroptosis. Review of extant literature indicates that kaempferol can attenuate neuronal damage caused by oxygen–glucose deprivation/reoxygenation and liver injury induced by acetaminophen via inhibiting ferroptosis by activating the Nrf2/SLC7A11/GPX4 signaling pathway [17, 18]. However, there is still a lack of evidence regarding kaempferol's ability to alleviate Aβ‐induced neuronal damage through the inhibition of neuronal ferroptosis. Our findings demonstrated that Aβ_1–42_‐induced increase in the levels of Fe^2+^ and MDA, accumulation of lipid ROS, and decrease in the levels of GSH/GSSG and SOD in PC12 cells were rescued following kaempferol treatment. Our data preliminarily revealed that kaempferol inhibited Aβ‐induced neuronal damage and improved the cognitive deficits of the AD mouse model through inhibition of ferroptosis.

The Xc¯/GPX4 pathway plays a crucial role in regulating ferroptosis. The Xc¯ system, which consists of SLC7A11 and SLC3A2, is involved in GSH synthesis [19]. The Nrf2/HO‐1 signaling pathway is also implicated in the production of GPX4. During ferroptosis, the activation of the Nrf2/HO‐1 signaling pathway and the translocation of Nrf2 to the nucleus are suppressed, leading to a decreased GPX4 level [20]. In addition, NQO1, AKR1C1, and AKR1C3, which are downstream targets of Nrf2, also show decreased expression during ferroptosis [21, 22, 23]. Our data confirmed that the expression of GPX4, NQO1, SLC7A11, AKR1C1, and AKR1C3 and the activity of the Nrf2/HO‐1 signaling pathway in the Aβ_1–42_‐induced PC12 cells or in the hippocampus tissues of AD mice were inhibited, but these effects were reversed with kaempferol treatment. The above data further support the role of kaempferol in inhibiting Aβ‐induced neuronal ferroptosis. We investigated the interactions between the targets of kaempferol and GPX4, NQO1, NFE2L2/Nrf2, HMOX1/HO‐1, SLC7A11, AKR1C1, and AKR1C3 and found that AKR1B1 may directly interact with multiple ferroptosis‐related proteins (such as GPX4, AKR1C3, Nrf2, and HO‐1). In our current study, in Aβ_1–42_‐treated neurons, the inhibition of AKR1B1 to the accumulation of Fe^2+^, MDA, and lipid ROS and the promotion of AKR1B1 to GSH synthesis and SOD activity were reversed by blocking the Nrf2 signaling pathway. Furthermore, increasing AKR1B1 facilitated the expression of GPX4 and AKR1C3 in Aβ_1–42_‐treated neurons, but this was counteracted by inhibiting the Nrf2 signaling pathway. We propose that AKR1B1 suppresses Aβ_1–42_‐induced neuronal ferroptosis by activating the Nrf2/HO‐1 signaling pathway, thereby promoting GPX4 and AKR1C3 expression. More importantly, we confirmed that the promotion of kaempferol to the activity Nrf2/HO‐1 and the expression of GPX4 and AKR1C3, and the suppression of kaempferol to ferroptosis in Aβ_1–42_‐treated neurons were partly reversed by silencing AKR1B1. Synapse loss, gliosis, and neuroinflammation are closely associated with AD. In the animal model, the effects of kaempferol in reducing Aβ and p‐Tau deposition, as well as neuroinflammation, were reversed by inhibiting AKR1B1.

## 5 Conclusion

Overall, kaempferol has the potential to prevent and inhibit Aβ‐triggered neuronal ferroptosis and ameliorate the cognitive impairment and pathological changes in the AD mouse model. These effects of kaempferol are achieved by increasing the expression of GPX4 and AKR1C3 through the upregulation of AKR1B1, which subsequently activates the Nrf2/HO‐1 signaling pathway. Our findings support the advancement of kaempferol for clinical application.

## Author Contributions

**Le Li:** conceptualization, data curation, investigation, methodology, software, writing – original draft, writing – review and editing. **Manying Yang:** conceptualization, methodology, formal analysis, validation, writing – original draft, writing – review and editing. **Jiale Tao:** data curation, investigation, visualization, writing – original draft. **Yonghong Zhao:** formal analysis, writing – original draft. **Shiguo Sun:** formal analysis, project administration, writing – review and editing. **Na Zhao:** formal analysis, supervision, writing – review and editing, methodology.

## Funding

This research was supported by the Corps Guiding Science and Technology Projects (No. 2022ZD050) and Scientific and technological research projects in key fields of the Corps (No. 2025DA013).

## Ethics Statement

All animal experiments were conducted with the approval of the Animal Experimentation Ethics Committee of Shihezi University and based on the ARRIVE guidelines.

## Consent

The authors have nothing to report.

## Conflicts of Interest

The authors declare no conflicts of interest.

## Supporting information


**Table S1:** The targets of kaempferol from different database.

**Table S2:** Primers used in real‐time qPCR.

## Data Availability

The data that support the findings of this study are available from the corresponding author upon reasonable request.
