# Supplementary material for: Kaempferol Improves Alzheimer's Disease by Inhibiting Neuronal Ferroptosis via Activating GPX4/AKR1C3 Signaling Pathway
Source: Pharmacol Res Perspect. 2026 Apr 30;14(3):e70255. doi: 10.1002/prp2.70255 (PMC13132798; doi:10.1002/prp2.70255)
Supplement: Supplementary file 2 — Table S2: Primers used in real‐time qPCR. [file PRP2-14-e70255-s001.docx]

| Table 2. Primers used in real-time quantitative PCR | | |
| --- | --- | --- |
| Gene | Forward (5′-3′) | Reverse (5′-3′) |
| rat Akr1b1 | CTCAACAACGGCACCAAGATG | CCATGTCGATAGCAACCTTCAC |
| rat Ar | TGGCGGTCCTTCACTAATGTCAAC | CATCCTCACGCACTGGCTGTAC |
| rat Cyp1b1 | CTCTCTGGAGAAATGGCCGA | AAAGGAAATCAAGCGCCACC |
| rat Glo1 | CGAGGGTTCTTGGACTGACG | TTCAGTGCCCCAGTTGTGTG |
| rat Alox5 | CCTACACTGTCACCGTAGCC | GACGTCATAGGAGTCCACCG |
| rat Gpx4 | ATTCCCGAGCCTTTCAACCC | CCCATCGATGTCCTTGGCTG |
| rat Nqo1 | TGAGCCCGGATATTGTAGCTGA | GCATACGTGTAGGCGAATCCTG |
| rat Slc7a11 | AACCCAAGTGGTTCAGACGATT | GGCAGATGGCCAAGGATTTGA |
| rat Akr1c1 | AGCCACCGTGAAACAAG | AGCCAGGACCACAACCC |
| rat Akr1c3 | TGAGGAGAGAATCAGAGAGA | TCAGAGAATGGAAAGTTAGG |
| rat Gapdh | AAGATGGTGAAGGTCGGTGT | AGGTCAATGAAGGGGTCGTT |
